# Supplementary material for: Mothers’ acceptability of using novel technology with video and audio recording during newborn resuscitation: A cross-sectional survey
Source: PLOS Digit Health. 2024 Apr 1;3(4):e0000471. doi: 10.1371/journal.pdig.0000471 (PMC10984542; doi:10.1371/journal.pdig.0000471)
Supplement: S1 Table — (DOCX) [file pdig.0000471.s001.docx]

**S1 Table. Reliability assessment of seven items using Cronbach’s alpha**

| **Cronbach's Alpha** | **N of Items** | **No. of cases** |
| --- | --- | --- |
| 0.859 | 7 | 21 |
