# Supplementary material for: Mothers’ acceptability of using novel technology with video and audio recording during newborn resuscitation: A cross-sectional survey
Source: PLOS Digit Health. 2024 Apr 1;3(4):e0000471. doi: 10.1371/journal.pdig.0000471 (PMC10984542; doi:10.1371/journal.pdig.0000471)
Supplement: S2 Table — (DOCX) [file pdig.0000471.s002.docx]

**S2 Table. Comparison of the socio-demographic characteristics between women who consented and those who did not consent.**

| **Respondent’s characteristics** | **Consent for the study** | | **χ2 statistics** | **p-value** |
| --- | --- | --- | --- | --- |
|  | **Yes** | **No** |  |  |
|  | **n (%)** | **n (%)** |  |  |
| **Age** | | | | |
| Up to 25 years | 11 (52.4) | 3 (100.0) | 2.449 | 0.118 |
| Above 25 years | 10 (47.6) | 0 (0.0) |  |  |
| **Education** | | | | |
| Illiterate (unable to read and write) | 1 (4.7) | 0 (0.0) | 17.143 | **0.004** |
| Up to primary level (Up to grade 5) | 0 (0.0) | 2 (66.6) |  |  |
| Up to secondary level (Up to grade 10) | 3 (14.3) | 1 (33.3) |  |  |
| Up to higher secondary level (SEE above) | 6 (28.6) | 0 (0.0) |  |  |
| Bachelor’s level | 6 (28.6) | 0 (0.0) |  |  |
| Master’s level or above | 5 (23.8) | 0 (0.0) |  |  |
| **Parity** | | | | |
| First baby (1) | 13 (61.9) | 2 (66.7) | 0.316 | 0.854 |
| Second baby (2) | 2 (9.5) | 0 (0.0) |  |  |
| More than 2 babies (3>) | 6 (28.6) | 1 (33.3) |  |  |
| **Ethnicity** | | | | |
| Brahmin/ Chhetri | 6 (28.5) | 0 (0.0) | 1.42 | 0.701 |
| Janajati | 9 (42.9) | 2 (66.7) |  |  |
| Madhesi | 1 (4.8) | 0 (0.0) |  |  |
| Dalit | 5 (23.8) | 1 (33.3) |  |  |
