# Supplementary material for: Mothers’ acceptability of using novel technology with video and audio recording during newborn resuscitation: A cross-sectional survey
Source: PLOS Digit Health. 2024 Apr 1;3(4):e0000471. doi: 10.1371/journal.pdig.0000471 (PMC10984542; doi:10.1371/journal.pdig.0000471)
Supplement: S3 Table — (DOCX) [file pdig.0000471.s003.docx]

**S3 Table.** **Acceptability by age (dichotomized based on median age of 25).**

| Variables | Total | Young Age (<25) | Older Age (>25) | p-value* |
| --- | --- | --- | --- | --- |
|  | (N =21) | (N=11) | (N=10) |  |
| ***I was comfortable with the baby’s care being video recorded*** |  |  |  | 0.36 |
| 1= Strongly Disagree | 0 | 0 | 0 |  |
| 2= Disagree | 0 | 0 | 0 |  |
| 3= Neutral/No Opinion | 2 (9.5) | 2 (18.2) | 0 |  |
| 4= Agree | 11 (52.4) | 5 (454) | 6 (60.0) |  |
| 5= Strongly Agree | 8 (38.1) | 4 (36.2) | 4 (40.0) |  |
| ***I was comfortable with someone using a tablet when observing my baby’s care*** |  |  |  | 0.62 |
| 1= Strongly Disagree | 0 | 0 | 0 |  |
| 2= Disagree | 0 | 0 | 0 |  |
| 3= Neutral/No Opinion | 1 (4.8) | 1 (9.0) | 0 |  |
| 4= Agree | 10 (47.6) | 5 (45.5) | 5 (50.0) |  |
| 5= Strongly Agree | 10 (47.6) | 5 (45.5) | 5 (50.0) |  |
| ***I* *was comfortable with someone observing the newborn resuscitation activity of my baby*** |  |  |  | 0.39 |
| 1= Strongly Disagree | 0 | 0 | 0 |  |
| 2= Disagree | 0 | 0 | 0 |  |
| 3= Neutral/No Opinion | 0 | 0 | 0 |  |
| 4= Agree | 10 (47.6) | 4 (36.4) | 6 (60.0) |  |
| 5= Strongly Agree | 11 (52.4) | 7 (63.6) | 4 (40.0) |  |
| ***Use of video and audio recording during resuscitation will neither cause harm nor will it compromise the care of my baby in the hospital*** |  |  |  | 0.51 |
| 1= Strongly Disagree | 0 | 0 | 0 |  |
| 2= Disagree | 0 | 0 | 0 |  |
| 3= Neutral/No Opinion | 1 (4.8) | 1 (9.1) | 0 |  |
| 4= Agree | 11 (52.4) | 6 (54.5) | 5 (50.0) |  |
| 5= Strongly Agree | 9 (42.9) | 4 (36.4) | 5 (50.0) |  |
| ***The MALA system will help to improve the health worker’s performance in newborn care*** |  |  |  | 0.41 |
| 1= Strongly Disagree | 0 | 0 | 0 |  |
| 2= Disagree | 0 | 0 | 0 |  |
| 3= Neutral/No Opinion | 1 (4.8) | 0 | 1 (10.0) |  |
| 4= Agree | 13 (61.9) | 8 (72.7) | 5 (50.0) |  |
| 5= Strongly Agree | 7 (33.3) | 3 (27.3) | 4 (40.0) |  |
| ***I trust that the information of my baby will be kept strictly confidential*** |  |  |  | 0.66 |
| 1= Strongly Disagree | 0 | 0 | 0 |  |
| 2= Disagree | 0 | 0 | 0 |  |
| 3= Neutral/No Opinion | 0 | 0 | 0 |  |
| 4= Agree | 13 (61.9) | 6 (54.5) | 7 (70.0) |  |
| 5= Strongly Agree | 8 (38.1) | 5 (45.5) | 3 (30.0) |  |
| ***I would recommend other mothers to participate in the MALA system*** |  |  |  | 0.83 |
| 1= Strongly Disagree | 0 | 0 | 0 |  |
| 2= Disagree | 0 | 0 | 0 |  |
| 3= Neutral/No Opinion | 7 (33.3) | 4 (36.4) | 3 (30.0) |  |
| 4= Agree | 7 (33.3) | 4 (36.4) | 3 (30.0) |  |
| 5= Strongly Agree | 7 (33.3) | 3 (27.2) | 4 (40.0) |  |

*Based on chi-square tests or Fisher’s exact test
