# Supplementary material for: Mothers’ acceptability of using novel technology with video and audio recording during newborn resuscitation: A cross-sectional survey
Source: PLOS Digit Health. 2024 Apr 1;3(4):e0000471. doi: 10.1371/journal.pdig.0000471 (PMC10984542; doi:10.1371/journal.pdig.0000471)
Supplement: S5 Table — (DOCX) [file pdig.0000471.s005.docx]

**S5 Table. Acceptability by education level (up to higher secondary level *vs.* bachelor’s level or higher).**

| Variables | Total | Up to higher secondary level | Bachelor’s level or higher | p-value |
| --- | --- | --- | --- | --- |
|  | (N =21) | (N=10) | (N=11) |  |
| ***I was comfortable with the baby’s care being video recorded*** |  |  |  | **0.026** |
| 1= Strongly Disagree | 0 | 0 | 0 |  |
| 2= Disagree | 0 | 0 | 0 |  |
| 3= Neutral/No Opinion | 2 (9.5) | 2 (20.0) | 0 |  |
| 4= Agree | 11 (52.4) | 7 (70.0) | 4 (36.4) |  |
| 5= Strongly Agree | 8 (38.1) | 1 (10.0) | 7 (63.6) |  |
| ***I was comfortable with someone using a tablet when observing my baby’s care*** |  |  |  | **0.046** |
| 1= Strongly Disagree | 0 | 0 | 0 |  |
| 2= Disagree | 0 | 0 | 0 |  |
| 3= Neutral/No Opinion | 1 (4.8) | 1 (10.0) | 0 |  |
| 4= Agree | 10 (47.6) | 7 (70.0) | 3 (27.3) |  |
| 5= Strongly Agree | 10 (47.6) | 2 (20.0) | 8 (72.7) |  |
| ***I* *was comfortable with someone observing the newborn resuscitation activity of my baby*** |  |  |  | 0.39 |
| 1= Strongly Disagree | 0 | 0 | 0 |  |
| 2= Disagree | 0 | 0 | 0 |  |
| 3= Neutral/No Opinion | 0 | 0 | 0 |  |
| 4= Agree | 10 (47.6) | 6 (60.0) | 4 (36.4) |  |
| 5= Strongly Agree | 11 (52.4) | 4 (40.0) | 7 (63.6) |  |
| ***Use of video and audio recording during resuscitation will neither cause harm nor will it compromise the care of my baby in the hospital*** |  |  |  | 0.1 |
| 1= Strongly Disagree | 0 | 0 | 0 |  |
| 2= Disagree | 0 | 0 | 0 |  |
| 3= Neutral/No Opinion | 1 (4.8) | 1 (10.0) | 0 |  |
| 4= Agree | 11 (52.4) | 7 (70.0) | 4 (36.4) |  |
| 5= Strongly Agree | 9 (42.9) | 2 (20.0) | 7 (63.6) |  |
| ***The MALA system will help to improve the health worker’s performance in newborn care*** |  |  |  | 0.31 |
| 1= Strongly Disagree | 0 | 0 | 0 |  |
| 2= Disagree | 0 | 0 | 0 |  |
| 3= Neutral/No Opinion | 1 (4.8) | 1 (10.0) | 0 |  |
| 4= Agree | 13 (61.9) | 7 (70.0) | 6 (54.5) |  |
| 5= Strongly Agree | 7 (33.3) | 2 (20.0) | 5 (45.5) |  |
| ***I trust that the information of my baby will be kept strictly confidential*** |  |  |  | 0.66 |
| 1= Strongly Disagree | 0 | 0 | 0 |  |
| 2= Disagree | 0 | 0 | 0 |  |
| 3= Neutral/No Opinion | 0 | 0 | 0 |  |
| 4= Agree | 13 (61.9) | 7 (70.0) | 6 (54.5) |  |
| 5= Strongly Agree | 8 (38.1) | 3 (30.0) | 5 (45.5) |  |
| ***I would recommend other mothers to participate in the MALA system*** |  |  |  | 0.83 |
| 1= Strongly Disagree | 0 | 0 | 0 |  |
| 2= Disagree | 0 | 0 | 0 |  |
| 3= Neutral/No Opinion | 7 (33.3) | 3 (30.0) | 4 (36.4) |  |
| 4= Agree | 7 (33.3) | 3 (30.0) | 4 (36.4) |  |
| 5= Strongly Agree | 7 (33.3) | 4 (40.0) | 3 (27.2) |  |
