# Supplementary material for: Mothers’ acceptability of using novel technology with video and audio recording during newborn resuscitation: A cross-sectional survey
Source: PLOS Digit Health. 2024 Apr 1;3(4):e0000471. doi: 10.1371/journal.pdig.0000471 (PMC10984542; doi:10.1371/journal.pdig.0000471)
Supplement: S6 Table — (DOCX) [file pdig.0000471.s006.docx]

**S6 Table. Acceptability by having a computer at home (yes vs. no).**

| Variables | Total | Yes | No | P-value |
| --- | --- | --- | --- | --- |
|  | (N =21) | (n=14) | (n=7) |  |
| ***I was comfortable with the baby’s care being video recorded*** |  |  |  | 0.28 |
| 1= Strongly Disagree | 0 | 0 | 0 |  |
| 2= Disagree | 0 | 0 | 0 |  |
| 3= Neutral/No Opinion | 2 (9.5) | 1 (7.1) | 1 (14.3) |  |
| 4= Agree | 11 (52.4) | 6 (42.9) | 5 (71.4) |  |
| 5= Strongly Agree | 8 (38.1) | 7 (50.0) | 1 (14.3) |  |
| ***I was comfortable with someone using a tablet when observing my baby’s care*** |  |  |  | 0.22 |
| 1= Strongly Disagree | 0 | 0 | 0 |  |
| 2= Disagree | 0 | 0 | 0 |  |
| 3= Neutral/No Opinion | 1 (4.8) | 0 | 1 (14.3) |  |
| 4= Agree | 10 (47.6) | 6 (42.9) | 4 (57.1) |  |
| 5= Strongly Agree | 10 (47.6) | 8 (57.1) | 2 (58.6) |  |
| ***I* *was comfortable with someone observing the newborn resuscitation activity of my baby*** |  |  |  | **0.024** |
| 1= Strongly Disagree | 0 | 0 | 0 |  |
| 2= Disagree | 0 | 0 | 0 |  |
| 3= Neutral/No Opinion | 0 | 0 | 0 |  |
| 4= Agree | 10 (47.6) | 4 (28.6) | 6 (85.7) |  |
| 5= Strongly Agree | 11 (52.4) | 10 (71.4) | 1 (14.3) |  |
| ***Use of video and audio recording during resuscitation will neither cause harm nor will it compromise the care of my baby in the hospital*** |  |  |  | 0.42 |
| 1= Strongly Disagree | 0 | 0 | 0 |  |
| 2= Disagree | 0 | 0 | 0 |  |
| 3= Neutral/No Opinion | 1 (4.8) | 1 (7.1) | 0 |  |
| 4= Agree | 11 (52.4) | 6 (42.9) | 5 (71.4) |  |
| 5= Strongly Agree | 9 (42.9) | 7 (50.0) | 2 (28.6) |  |
| ***The MALA systemwill help to improve the health worker’s performance in newborn care*** |  |  |  | 0.7 |
| 1= Strongly Disagree | 0 | 0 | 0 |  |
| 2= Disagree | 0 | 0 | 0 |  |
| 3= Neutral/No Opinion | 1 (4.8) | 1 (7.1) | 0 |  |
| 4= Agree | 13 (61.9) | 8 (57.1) | 5 (71.4) |  |
| 5= Strongly Agree | 7 (33.3) | 5 (35.7) | 2 (28.6) |  |
| ***I trust that the information of my baby will be kept strictly confidential*** |  |  |  | 0.17 |
| 1= Strongly Disagree | 0 | 0 | 0 |  |
| 2= Disagree | 0 | 0 | 0 |  |
| 3= Neutral/No Opinion | 0 | 0 | 0 |  |
| 4= Agree | 13 (61.9) | 7 (50.0) | 6 (85.7) |  |
| 5= Strongly Agree | 8 (38.1) | 7 (50.0) | 1 (14.3) |  |
| ***I would recommend other mothers to participate in the MALA system*** |  |  |  | 0.22 |
| 1= Strongly Disagree | 0 | 0 | 0 |  |
| 2= Disagree | 0 | 0 | 0 |  |
| 3= Neutral/No Opinion | 7 (33.3) | 3 (21.4) | 4 (57.1) |  |
| 4= Agree | 7 (33.3) | 5 (35.7) | 2 (28.6) |  |
| 5= Strongly Agree | 7 (33.3) | 6 (42.9) | 1 (14.3) |  |
