# Supplementary material for: Mothers’ acceptability of using novel technology with video and audio recording during newborn resuscitation: A cross-sectional survey
Source: PLOS Digit Health. 2024 Apr 1;3(4):e0000471. doi: 10.1371/journal.pdig.0000471 (PMC10984542; doi:10.1371/journal.pdig.0000471)
Supplement: Attachment — Submitted filename: Response to the Reviewer_PLOS digital_03.10.23.docx [file pdig.0000471.s008.docx]

**Mothers’ acceptability of using novel technology with video and audio recording during**

**newborn resuscitation: A cross-sectional survey**

**Response to First Reviewer’s comment**

**Comment #1:** Studied sample size is very low, so any conclusion regarding the mother's acceptability for the video recording of their newborn's resuscitation couldn't be drawn.

Response- We agree to the comment, due to the sample size the study cannot provide recommendation that mothers acceptability. However, this is a piloting of the video recording of newborn resuscitation, before launching a full phase trial. The results of the study will provide research team on how to improve acceptability of mothers towards the technology. Therefore, we have previously acknowledged this limitation in the discussion and have further emphasized as follow in the Discussion section:

“Second, our results could not be generalized to other institutions or countries since this was a single-site study with a small sample size and parents are often not allowed to at the bedside during resuscitation or observe resuscitation procedures in low-resource settings. Therefore, study results and conclusion should be interpreted carefully. Third, sample size was not adequate to assess the difference in acceptability by the maternal education. A survey with adequate sample size, will be required to come to the conclusion that there was difference in acceptability rate by maternal education.”

**Comment #2**. Also, the studied mothers were selected on Voluntary basis so acceptability of the technology in mothers has not been properly established.

Response- We have now added the paragraph in line 123-131. “All the mothers, who were eligible to the study, were approached by the research team. Those mothers who agreed to be part of the study on using the video recording for resuscitation were included in the study and thus the technology was used among the consented mothers. Further, after the video recording of the resuscitation events, mothers were again approached whether they consent the video recording to be used for research purpose.”

**Comment #3.** The study needs to be evaluated, also in mothers having elementary level of education, as per the findings mothers with lower education level were not so comfortable with the technology, so proper survey needs to be done.

Response- We agree that the sample size is not adequate to assess the difference in acceptability by the maternal education. A survey with adequate sample size, will be required to come to the conclusion that there was difference in acceptability rate by maternal education. We have now added this in the limitation section. We have added in it in lines 337-340.

**Response to second Reviewer’s comment**

**Comment #4:** Interesting paper with a solid methodological basis and reporting of findings. It would benefit from proofreading and some sections in the results can be condensed to as it is reported in the tables.

Response- We have now done the proof reading of the paper and reporting of the results.

**Response to third Reviewer’s comment**

**Comment #5:** First of all, thank you for doing work on trying to improve newborn resuscitation. I believe this is a valuable research area and I hope that you continue working to develop MALA. However, I believe the current state of the project is not ready for publication given the type of analysis performed in this study.

Response- As mentioned to the first reviewer’s comment as well as in the manuscript, this is a pilot study among a limited number of infants who had resuscitation and ventilation. Though a sample size of 21 infants who were ventilated seems to be small, however, incidence rate of infants requiring ventilation is 1.6%, indicating that to have the sample size of 21 infants with ventilation 1375 infants needs to be observed. For observing a larger sample size of infants to enroll more than 100 infants with ventilation, this study was required to be assured that mother accept the video recording of the rare but critical events.

**Comment #6:** 106-107: There may be several other reasons for MALA to not be accepted by mothers, such as perceived lack of skill from HCPs due to reliance on the technology, lack of trust in technology to guide life saving actions, etc. This paper primarily only tackles the recording aspect, which the authors have already pointed out has been investigated before with varying results (315 - 322). Authors also mention this limitation on 328-330.

Response- This study was done with the pre-notion that mothers are not used to video recording of their resuscitation events and we made the assumptions that mothers might not accept the technology, hence conducted the study (mentioned in lines 106-107). Previous studies conducted in high income setting to access the parent’s acceptability to video recording showed concerns of recording for future use as well as requested for video. We have elaborated now in lines 364-366 as “ study showed that mothers were comfortable with video recording and indicated that the video display might improve the health care provider’s performance”.

In the limitation, 350-352, we had mentioned that “…since this study evaluated mothers’ acceptability of the first phase of the MALA system without real-time automated feedback, further studies evaluating acceptability of the complete MALA system are warranted.”, we have now further elaborated it as “the study assessed the acceptability of video recording with display of timing of ventilation in the resuscitation monitor, which was accepted by the mother. However, the MALA system is further being developed with audio feedback to health care provider on steps of resuscitation. This further improvised MALA system needs to be assessed for acceptability of the parents”

**Comment #7:** 229-230: Questions are a little leading. For example, for “I was comfortable with the baby’s care being video recorded” could have been “How did you feel about the baby’s care being video recorded” and have “uncomfortable”, “slightly uncomfortable”, "neutral”, “fairly comfortable”, comfortable”. This makes me question the reliability of the results.

Response- We have now run a reliability test on the questions in place using the Cronbach’s Alpha statistics which showed that the values of each score was more than >0.4. We have kept it in the supplementary file.

**Comment #8:** 310-312: The lack of information about why mothers were hesitant to recommend this novel technology to other mothers makes the contribution of this paper weaker. I do not see how this submission could influence the future direction of the development of the technology or how impact how to more effectively get mothers to accept the technology. Given that other publications that the authors point out have also looked into the acceptance of video recording infants, the contribution of this paper seems limited.

Response- We agree that mothers were hesitant recommending the technology to other mothers, which provides a valuable information to the technology development team and research team. There is a need to further refine the technology through a co-design process with the mothers and caregivers, so that it is acceptable to mothers. The other papers we refer to in the manuscripts were from the high-income settings where health literacy of mothers is different than the mothers from the study site. Thus, valuable information from the study provides two major learnings for this research and other research which recording sensitive as well as critical data point on immediate newborn care. First, mothers should be engaged in the development of the technology, so that they accept the agronomics as well as the intent of the technology and second, there is a need to improve the maternal health literacy of the video and audio recording of the immediate newborn care. We have now added this in our conclusion statement. We have now added it in lines 354-363.

**Comment #9:** 323-325: The bias from only having data for individuals who participated in the study is a big concern, especially considering that there were 3 deliveries where the mothers did not consent to participate in the survey and their positions are unrepresented here.

Response- We acknowledge among the women who did not participate might be unrepresentative of the women who consented and thus might create a bias in terms of representation of the population of women coming to the hospital for childbirth unless a comparison is done. For this, we have now compared the socio-demographic (age, ethnicity, education, parity) of the women who participated vs who did not participate using Pearson’s chi-square test (Supplementary Table 2).
